# miR-127-3p regulates osteoporosis progression by targeting VAMP2 to modulate cell differentiation

**DOI:** 10.1530/JOE-25-0032

**Published:** 2025-08-21

**Authors:** Jing Chen, Jing Lei, Ganggang Wang, Huiling Qin, Li Yang

**Affiliations:** ^1^ Department of Endocrinology and Metabolism, Shaoxing People’s Hospital, Shaoxing, China; ^2^ The First Affiliated Hospital, Shaoxing University, Shaoxing, China; ^3^ Department of Radiology, The Seventh Affiliated Hospital of Sun Yat-sen University, Shenzhen, Guangdong, China; ^4^ Department of Hand and Foot Surgery, Zhucheng People’s Hospital, Weifang, Shandong, China; ^5^ Department of Rehabilitation, The Affiliated Hospital of Youjiang Medical University for Nationalities, Baise, China; ^6^ Guangxi Key Laboratory for Preclinical and Translational Research on Bone and Joint Degenerative Diseases, Baise, Guangxi, China; ^7^ Department of Orthopaedics, Chongqing Public Health Medical Center, Chongqing, China

**Keywords:** miR-127-3p, VAMP2, osteoporosis, human bone mesenchymal stem cells, differentiation

## Abstract

Osteoporosis (OP) is a systemic osteopathy characterized by a decrease in bone density and mass. Human bone mesenchymal stem cells (hBMSCs) exhibit multidirectional differentiation potential and play a critical role in bone metabolism. Herein, we investigated the diagnostic potential of miR-127-3p in OP and elucidated its regulatory role in hBMSCs, thereby providing novel insights into the diagnosis and progression prediction of OP. The relative expression of miR-127-3p was measured via RT-qPCR analysis. ROC curve and logistic analysis were applied to identify the diagnostic value of miR-127-3p for OP. The CCK8 assay and flow cytometry were used to assess cell viability and apoptosis. A luciferase reporter assay was performed to assess the interaction between VAMP2 and miR-127-3p. The bone differentiation markers RUNX2, OCN, and OPN were assayed using RT-qPCR and western blotting. We observed that the expression of miR-127-3p was reduced in patients with OP, allowing it to effectively distinguish these patients from healthy individuals. Transfection with miR-127-3p mimic inhibited hBMSC apoptosis, increased cell viability, and increased RUNX2, OCN, and OPN levels. Furthermore, miR-127-3p regulated hBMSCs via targeting VAMP2. Overexpression of VAMP2 reversed the effects of miR-127-3p on apoptosis, cell viability, and bone differentiation. In conclusion, these findings suggest that miR-127-3p may be a potential diagnostic tool for OP. In addition, miR-127-3p promotes hBMSC viability and differentiation through downregulating VAMP2; this decreases OP progression. Our findings will inform new ideas for the diagnosis and developmental prediction of OP.

## Introduction

Osteoporosis (OP) is a common systemic and metabolic skeletal disease. It is characterized by reduced bone mass, heightened fragility, destruction of bone tissue microstructure, and an increased propensity for fragility fractures (Aibar-Almazán *et al.* 2022). OP is common in postmenopausal women and men over the age of 50. Recently, along with the aging of the Chinese population, the incidence of OP has shown a rising trend. This has significantly increased the affected population and imposed a substantial economic and social burden (Gao *et al.* 2023). Human bone mesenchymal stem cells (hBMSCs) are mesodermal pluripotent stem cells with multidirectional differentiation potential and have an essential function in bone formation and metabolism (Ni *et al.* 2020). In patients with OP, the osteogenic differentiation level and bone density are lower (Niedermair *et al.* 2020). The senescence of hBMSCs and their impaired osteogenic differentiation are thought to be closely associated with OP, and increasing the activity and number of BMSCs can improve OP (Tian *et al.* 2021). Therefore, promoting the differentiation of hBMSCs to osteoblasts could be a novel approach to OP treatment.

MicroRNAs (miRNAs) are allowed to bind to target genes through base pairing, leading to their degradation or translational repression (Pepin & Gantier 2016). miRNAs can participate in bone formation by targeting and regulating multiple transcription elements in stem cells, osteoblasts, osteoclasts, and chondrocytes (Taipaleenmäki 2018). Osteoblast differentiation is an essential procedure for bone reconstruction in OP. Hence, miRNAs regulate the biological process of osteoblast differentiation and may serve as potential therapeutic targets or biomarkers for OP (Yang *et al.* 2020). Increasing evidence suggests that miRNAs can regulate the differentiation of hBMSCs to osteoblasts. For example, miR-224-5p can regulate osteogenic differentiation by targeting RUNX2 (Ding *et al.* 2023). miR-485-5p can regulate hBMSC differentiation to osteoblasts by targeting osterix, presenting a potential therapeutic strategy for OP (Zhang *et al.* 2018*b*). Previous studies have revealed that miR-127-3p is involved in the proliferation and metastasis of multiple cancers, such as ovarian cancer and renal cell carcinoma (Du *et al.* 2020, Song *et al.* 2024). Recent research has revealed the role of miR-127-3p in cell differentiation and the regulation of orthopedic diseases. In particular, it participates in fibroblast differentiation and regulates skin repair functions (Auler *et al.* 2021). Dong *et al.* have reported that miR-127-3p can alleviate osteoarthritis by regulating CDH11 (Dong *et al.* 2021). In addition, high-throughput sequencing and bioinformatics analyses suggest that miR-127-3p expression is downregulated in patients with OP and femoral head necrosis (Hong *et al.* 2019, Huang *et al.* 2024). Nevertheless, the mechanism of action of miR-127-3p in OP remains unknown.

Vesicle-associated membrane protein 2 (VAMP2) was originally discovered in the synaptic vesicles of the rat brain and participated in multiple cellular regulation processes (Baumert *et al.* 1989). Some studies of VAMP2 related to human bone have been reported; for example, miR-185 can inhibit the proliferation of osteosarcoma cells by targeting VAMP2 (Li *et al.* 2019). Moreover, VAMP2 has been identified as a hub gene in postmenopausal OP (Pala & Denkçeken 2019). However, the association of miR-127-3p with VAMP2 in OP remains unclear.

In the present study, we investigated the diagnostic ability of miR-127-3p for OP and its function and possible mechanisms in regulating the apoptosis, cell viability, and differentiation of hBMSCs. This study provides prospects of miR-127-3p as a biomarker for the diagnosis and treatment of OP.

## Materials and methods

### Study objects and data collection

The protocol of this study was endorsed by the Ethics Committee of Zhucheng People’s Hospital and informed consent was executed with those included into study. The sample size was calculated based on the G-power software. When the alpha error probability was 0.05 and the p-power (1-beta) was 0.95, it was estimated that the necessary sample size for each group was more than 88. Therefore, this study retrospectively included 90 patients who came to our hospital for the treatment or diagnosis of OP in 2021–2023. The control group included 90 healthy individuals who simultaneously attended physical check-ups at the hospital. Inclusion criteria were as follows: individuals aged 55–75 years; those who met the diagnostic criteria for OP (Rentzeperi *et al.* 2023); and those without a history of prior anti-OP treatment. Exclusion criteria are as follows: patients with malignant tumors, severe hepatic or renal insufficiency, or immune system diseases; those with concomitant metabolic bone diseases such as osteochondrosis; and those who had recently taken hormonal drugs.

General information such as age, gender, and body mass index (BMI) was collected from the patient’s electronic medical record. Bone mineral density (BMD) was measured by X-ray. Fasting venous blood was taken from participants and sent to our laboratory to measure serum 25(OH)D, calcium, phosphorus, and alkaline phosphatase (ALP) levels.

### Cell culture and differentiation

hBMSCs were procured from the Chinese Academy of Sciences (Shanghai, China) and cultured in DMEM (Thermo, USA) containing 10% fetal bovine serum and 1% penicillin–streptomycin. Differentiation of hBMSCs was induced using β-glycerophosphate (4 mmol/L) and ascorbic acid (25 mg/L). Cells were incubated in a 37°C incubator containing 5% CO_2_.

### Real-time quantitative polymerase chain reaction (RT-qPCR) detection

Total RNA was purified from serum or hBMSCs using Trizol reagent (Invitrogen, USA). Briefly, 1 mL Trizol reagent was added to centrifuge tubes spiked with serum or hBMSCs, and it was left at room temperature for 5 min for complete lysis. Subsequently, 200 μL chloroform was added, the mixture was shaken well, and it was allowed to stand for 3 min. Thereafter, the tubes were centrifuged at 12,000 ***g*** for 10 min in a centrifuge at 4°C. The supernatants were pipetted into new centrifuge tubes, followed by the addition of 500 μL isopropanol. The tubes were allowed to stand for 10 min and then centrifuged at 12,000 ***g*** at 4°C for 10 min. Later, the supernatant was discarded and the pellet was washed twice with 75% ethanol. After drying the precipitate, DEPC water was added to dissolve the RNA. RNA concentration and purity were assayed using NanoDrop 2000 (Invitrogen, USA). Subsequently, RNA was reverse transcribed to cDNA using PrimeScript RT Kit (Takara, Japan). RT-qPCR reactions were performed according to the manufacturer’s instructions for the SYBR Green PCR Master Mix Kit (Invitrogen, USA). The 2^−ΔΔCt^ method was used to calculate the relative expression of miR-127-3p, VAMP2, RUNX2, OCN, and OPN, normalized to GAPDH and U6. The primers in this study are listed in Table 1.

**Table 1:** PCR primers in this study.

	Primers (5′-3′)
miR-127-3p	Forward: GCC​GAG​UCG​GAU​CCG​UCU​GA
	Reverse: CTCAACTGGTGTCGTGGA
VAMP2	Forward: GGTCTCTCCTGCGTTCCC
	Reverse: ACACTGGGTCCGCTCCTT
RUNX2	Forward: GGA​GCG​GAC​GAG​GCA​AGA​GT
	Reverse: AGG​AAT​GCG​CCC​TAA​ATC​AC
OCN	Forward: CAG​CGA​GGT​AGT​GAA​GAG​AC
	Reverse: GGA​TTC​CTG​GCT​GAC​TTT​GGA
OPN	Forward: CAC​TCC​AAT​CGT​CCC​TAC​AG
	Reverse: CCT​TAG​ACT​CAC​CGC​TCT​TC
GAPDH	Forward: CAT​CAA​CGG​GAA​GCC​CAT​C
	Reverse: CTC​GTG​GTT​CAC​ACC​CAT​C
U6	Forward: CTCGCTTCGGCAGCACA
	Reverse: AAC​GCT​TCA​CGA​ATT​TGC​GT

### Cell transfection

On the afternoon of the day before transfection, hBMSCs were digested with trypsin after washing with PBS. After centrifugation, the cells were resuspended and seeded in 6-well plates. The cell density was approximately 5 × 10^4^/mL. The cell fusion reached about 70% within 24 h, which could be used for transfection. miR-127-3p mimic, miR-127-3p inhibitor, mimic negative control (mimic NC), inhibitor negative control (inhibitor NC), overexpression VAMP2 (oe-VAMP2), silenced VAMP2 (si-VAMP2), overexpression negative control (oe-NC), and silenced negative control (si-NC) were synthesized by GenePharma (China). Referring to the instructions for Lipofectamine 2000, they were transfected into hBMSCs.

### CCK8 assay

The transfected hBMSCs were seeded into 96-well plates and incubated at 37°C and 5% CO_2_ to examine proliferation. The cells were treated at 0, 24, 48, and 72 h with 10 μL CCK8 reagent (Sigma Aldrich, USA). After incubation for 2 h, the OD value at 450 nm was determined using Microplate Reader (Thermo, USA).

### Apoptosis assay

The transfected hBMSCs were gathered and suspended in PBS. Then, Annexin V-FITC (Absin, China) and propidium iodide were added for staining. The cells were placed in a dark room at 20–25°C for 10–20 min, followed by the assessment of apoptosis using flow cytometry (BD, USA).

### Western blot

Protein samples were prepared by treating cells with 100 μL RIPA lysate (Beyotime, China) containing 1% protease inhibitor (Merck, USA). The BCA kit (Thermo, USA) was used to detect protein concentration. Denatured proteins were separated using 10% SDS-PAGE. The separated proteins were transferred to a PVDF membrane, which was washed with TBS and blocked with 5% skimmed milk powder for 1 h. Then, primary antibodies (Abcam, USA) were added, followed by overnight incubation at 4°C. On the following day, secondary antibodies (Abcam, USA) were added and incubated at 4°C for 4 h. Antibodies against GAPDH (Abcam, Britain) were utilized as a control. ECL system and ImageJ were used to visualize and analyze the protein blots.

### Luciferase reporter assay

The target genes of miR-127-3p were forecasted using the TargetScan7.2. The 3′ UTRs of wild-type and mutant VAMP2 were cloned into luciferase reporter plasmids to generate VAMP2-WT or VAMP2-MUT, respectively. The luciferase reporter vector and miR-127-3p mimic or inhibitor were co-transfected into hBMSCs using Lipofectamine 2000 (Invitrogen, USA). After 48 h, luciferase activity was assayed using a dual luciferase reporter gene assay system (Promega, USA) with Renilla as the reference.

### Statistical analysis

All data were analyzed using GraphPad Prism 9.0 and SPSS 27.0, and all values were presented as the mean ± SD. Statistical analyses were calculated using Student's *t*-test and one-way ANOVA. Diagnostic accuracy of miR-127-3p in patients with OP was analyzed by ROC curve. Each test was repeated at least three times and values were considered significant when the *P*-value was less than 0.05.

## Results

### Diagnostic value of miR-127-3p for OP

A total of 90 patients with OP and 90 healthy individuals were included in the study and their general information was contrasted in Table 2. Obviously, there was no significant difference between the OP and healthy groups in terms of age and BMI; however, the gender exhibited a meaningful difference (*P* = 0.037). In addition, other clinical indicators such as BMD, 25(OH)D, calcium and phosphorus were lower in the OP group than in the healthy group and the difference was significant (*P* < 0.001). Conversely, the serum ALP level was considerably higher in patients with OP than in healthy individuals (*P* < 0.001).

**Table 2:** General information on the subjects.

Parameters	Subjects		*P* value
	Healthy (*n* = 90)	Osteoporosis (*n* = 90)	
Age (years), mean ± SD	65.46 ± 5.54	66.48 ± 6.16	0.243
Gender (male/female)	49/41	35/55	0.037
BMI (kg/m^2^), mean ± SD	23.57 ± 3.09	23.12 ± 2.89	0.313
BMD			
LBMD (g/cm^2^), mean ± SD	0.99 ± 0.13	0.80 ± 0.12	<0.001
TBMD (g/cm^2^), mean ± SD	0.95 ± 0.12	0.77 ± 0.12	<0.001
25(OH)D (ng/mL), mean ± SD	31.23 ± 3.17	23.02 ± 3.11	<0.001
Calcium (mmol/L), mean ± SD	2.44 ± 0.19	2.30 ± 0.21	<0.001
Phosphorus (mmol/L), mean ± SD	1.31 ± 0.16	1.13 ± 0.15	<0.001
ALP (U/L), mean ± SD	122.19 ± 14.98	139.57 ± 15.90	<0.001

BMI, body mass index; BMD, bone mineral density; LBMD, lumbar bone mineral density; TBMD, total hip bone mineral density; 25(OH)D, 25-hydroxyvitamin D; ALP, alkaline phosphatase. All data were presented as the mean ± standard deviation or *n*.

**Figure 1:**
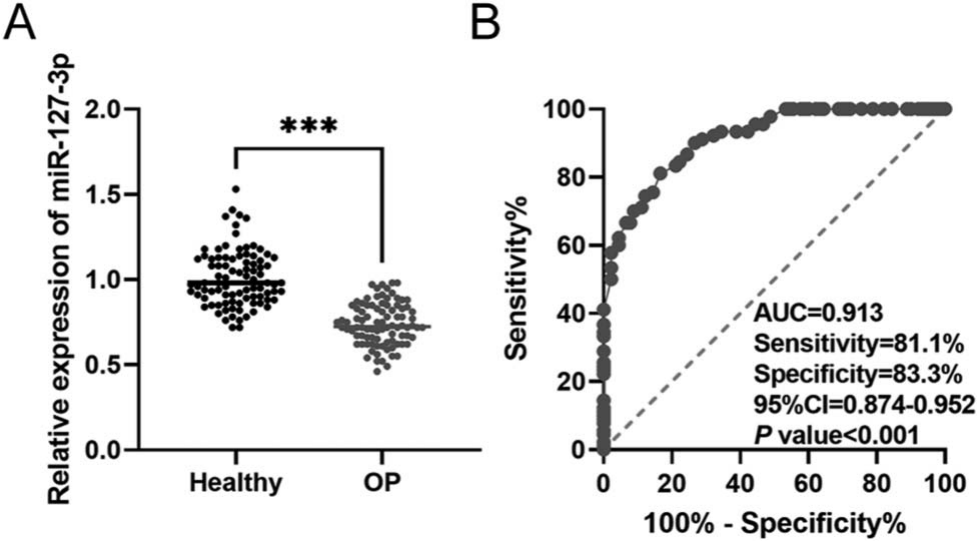
Diagnostic value of miR-127-3p for OP. (A) miR-127-3p expression in patients with OP and healthy individuals. (B) ROC curve for the diagnostic accuracy of miR-127-3p in patients with OP. ***P < 0.001.

To assess the diagnostic value of miR-127-3p in patients with OP, we measured serum miR-127-3p levels in OP and healthy groups. miR-127-3p expression was remarkably lower in the OP group than in the healthy group (***P < 0.001, Fig. 1A). ROC curve analysis revealed high sensitivity (81.1%) and specificity (83.3%) of miR-127-3p for diagnosing OP, with an AUC of 0.913 (95% CI = 0.874–0.952, *P* < 0.001, Fig. 1B). In addition, logistic analysis revealed that miR-127-3p is a pivotal element in inducing the pathogenesis of OP (*P* < 0.001). Furthermore, LBMD, TBMD, 25(OH)D, phosphorus, and ALP were significantly correlated with OP occurrence (*P* < 0.05, Table 3).

**Table 3:** Logistic regression analysis of factors associated with osteoporosis.

Parameters	OR	95% CI	*P*
miR-127-3p	0.030	0.007–0.126	<0.001
Gender (male/female)	0.419	0.125–1.406	0.159
LBMD (g/cm^2^)	0.168	0.050–0.570	0.004
TBMD (g/cm^2^)	0.138	0.040–0.481	0.002
25(OH)D (ng/mL)	0.203	0.057–0.715	0.013
Calcium (mmol/L)	0.333	0.085–1.308	0.115
Phosphorus (mmol/L)	0.168	0.048–0.590	0.005
ALP (U/L)	3.946	1.205–12.917	0.023

OR, odds ratio; 95% CI, 95% confidence interval; BMI, body mass index; BMD, bone mineral density; LBMD, lumbar bone mineral density; TBMD, total hip bone mineral density; 25(OH)D, 25-hydroxyvitamin D; ALP, alkaline phosphatase.

### Effect of miR-127-3p on the apoptosis and viability of hBMSCs

**Figure 2:**
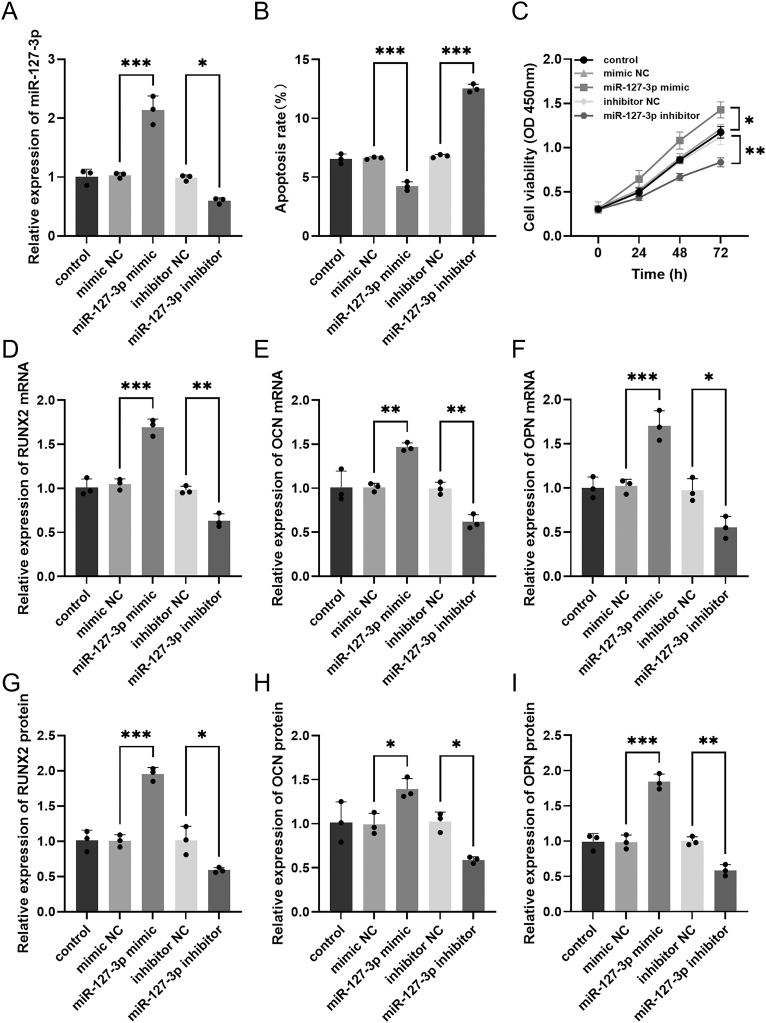
Effect of miR-127-3p on hBMSCs. Effect of up- or downregulation of miR-127-3p on (A) miR-127-3p expression, (B) apoptosis, (C) cell viability, (D, E, F) the expression of the bone differentiation markers RUNX2, OCN, and OPN, and (G, H, I) the protein levels of RUNX2, OCN and OPN *P < 0.05, **P < 0.01, ***P < 0.001.

To evaluate the effect of miR-127-3p on the apoptosis and proliferation of hBMSCs, cells were transfected with either miR-127-3p mimics or inhibitors. RT-qPCR was applied to assess miR-127-3p expression in hBMSCs. miR-127-3p mimic and miR-127-3p inhibitor upregulated and downregulated miR-127-3p expression, respectively (*P < 0.05, ***P < 0.001, Fig. 2A). The apoptosis outcomes demonstrated that transfection with miR-127-3p mimic slowed down apoptosis rate, while transfection with miR-127-3p inhibitor facilitated apoptosis (***P < 0.001, Fig. 2B). The CCK8 assay revealed that miR-127-3p mimic notably enhanced cell viability, while miR-127-3p inhibitor markedly reduced cell viability. Finally, the mimic and inhibitor negative controls did not affect cell viability (*P < 0.05, **P < 0.01, Fig. 2C).

### Effects of miR-127-3p on the differentiation of hBMSCs

To estimate the role of miR-127-3p in hBMSC differentiation, we assayed the levels of the bone differentiation markers RUNX2, OCN, and OPN in cells after transfection with either a miR-127-3p mimic or a miR-127-3p inhibitor, at both mRNA and protein levels. The results from RT-qRCR revealed that overexpression of miR-127-3p increased RUNX2, OCN, and OPN levels, while inhibition of miR-127-3p remarkably decreased RUNX2, OCN, and OPN levels (*P < 0.05, **P < 0.01, ***P < 0.001, Fig. 2D, E, F). At the protein level, RUNX2, OCN, and OPN exhibited a similar result (*P < 0.05, **P < 0.01, ***P < 0.001, Fig. 2G, H, I).

### miR-127-3p targets VAMP2 in hBMSCs

**Figure 3:**
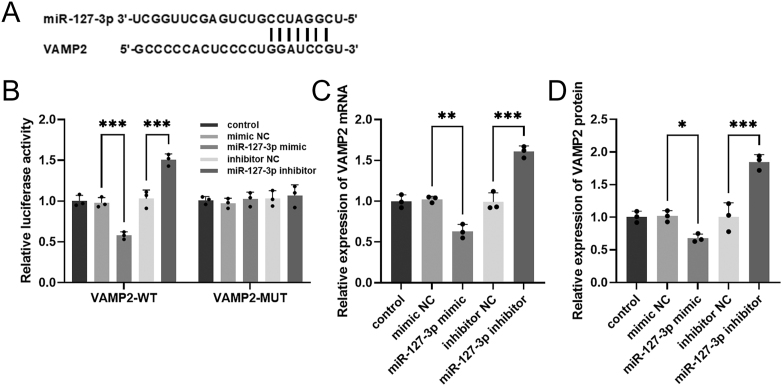
miR-127-3p targets VAMP2 in hBMSCs. (A) Binding site of miR-127-3p with VAMP2. (B) Luciferase activity of VAMP2-WT and VAMP2-MUT in hBMSCs transfected with miR-127-3p mimic or miR-127-3p inhibitor. (C and D) Effect of transfection with miR-127-3p mimic or miR-127-3p inhibitor on the mRNA and protein levels of VAMP2 in hBMSCs. *P < 0.05, **P < 0.01, ***P < 0.001.

We observed that miR-127-3p binds to the 3′UTR region of VAMP2 (Fig. 3A). The luciferase reporter assay suggested that the overexpression and inhibition of miR-127-3p decreased and increased the luciferase activity of VAMP2-WT, respectively. However, transfection with miR-127-3p mimic or miR-127-3p inhibitor did not affect the luciferase activity of WAMP2-MUT (***P < 0.001, Fig. 3B). In addition, our results further indicated that miR-127-3p overexpression inhibited VAMP2 levels dramatically, whereas inhibition of miR-127-3p promoted VAMP2 levels (**P < 0.01, ***P < 0.001, Fig. 3C). Western blotting revealed that transfection of miR-127-3p mimic decreased VAMP2 protein levels, while transfection of miR-127-3p inhibitor showed an opposite result (*P < 0.05, ***P < 0.001, Fig. 3D).

### Effects of VAMP2 on hBMSCs

**Figure 4:**
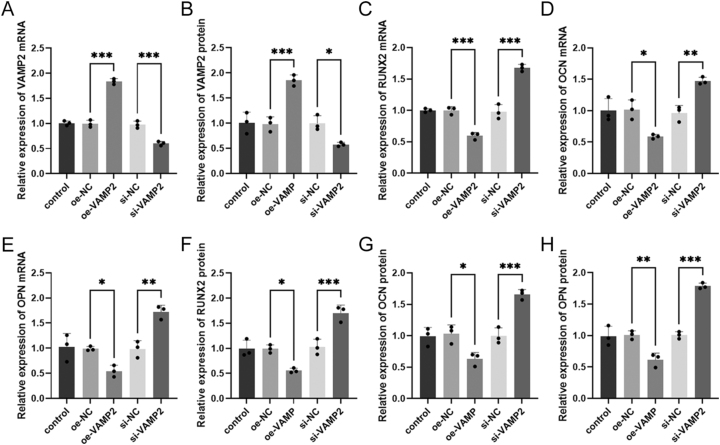
Effects of VAMP2 on hBMSCs. Effect of VAMP2 overexpression or silencing on (A) VAMP2 expression, (B) VAMP2 protein levels, (C, D, E) expression of the bone differentiation markers RUNX2, OCN, and OPN, and (F, G, H) the protein levels of RUNX2, OCN and OPN. *P < 0.05, **P < 0.01, ***P < 0.001.

To further determine the role of VAMP2 in the differentiation of hBMSCs, we analyzed its effect on bone differentiation markers by overexpressing or silencing VAMP2. RT-qPCR and western blotting revealed that overexpressing or silencing VAMP2 were able to, respectively, raise or reduce the VAMP2 mRNA and protein levels (*P < 0.05, ***P < 0.001, Fig. 4A and B). Overexpression of VAMP2 remarkably decreased RUNX2, OCN, and OPN levels, whereas VAMP2 silencing caused an opposite trend in RUNX2, OCN, and OPN levels (*P < 0.05, **P < 0.01, ***P < 0.001, Fig. 4C, D, E). In addition, the upregulation of VAMP2 decreased the protein levels of bone differentiation markers, whereas the downregulation of VAMP2 elevated their levels (*P < 0.05, **P < 0.01, ***P < 0.001, Fig. 4F, G, H).

### miR-127-3p regulates hBMSC apoptosis and viability by inhibiting VAMP2

**Figure 5:**
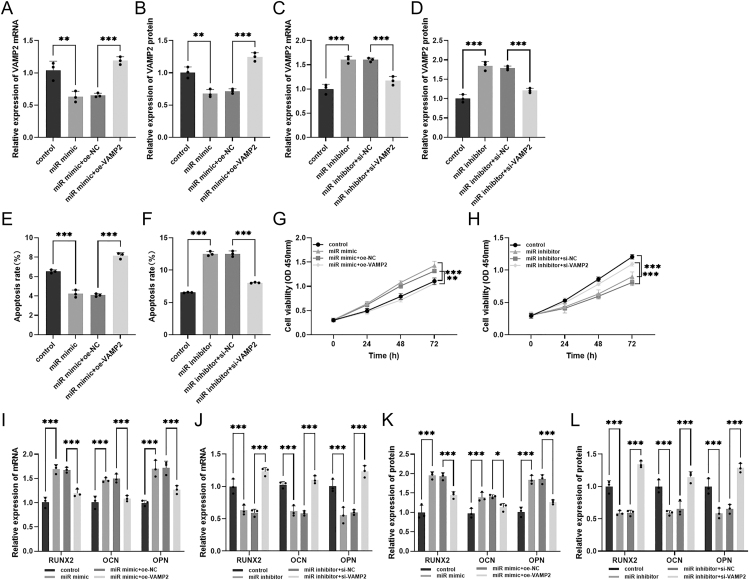
miR-127-3p regulates hBMSCs by inhibiting VAMP2. Effect of co-transfection with miR-127-3p mimic and oe-VAMP2 or miR-127-3p inhibitor and si-VAMP2 on (A, B, C, D) VAMP2 expression and protein levels, (E and F) apoptotic cells, (G and H) cell viability, and (I, J, K, L) the mRNA and protein levels of the bone differentiation markers RUNX2, OCN, and OPN. *P < 0.05, **P < 0.01, ***P < 0.001.

We further explored the actions of miR-127-3p and VAMP2 on the apoptosis, proliferation, and osteogenic differentiation of hBMSCs. The upregulation of miR-127-3p reduced VAMP2 expression and protein level, whereas the overexpression of VAMP2 ameliorated the suppressive effect of miR-127-3p (**P < 0.01, ***P < 0.001, Fig. 5A and B). Similarly, transfection with miR-127-3p inhibitor facilitated VAMP2 expression and protein levels, whereas silencing of VAMP2 reversed this trend (***P < 0.001, Fig. 5C and D). Analysis of apoptosis revealed that miR-127-3p overexpression inhibited apoptosis, while miR-127-3p downregulation showed an opposite tendency. However, co-transfection outcomes demonstrated that VAMP2 reverses the apoptosis effect of miR-127-3p (***P < 0.001, Fig. 5E and F). The CCK8 assay revealed that miR-127-3p mimic elevated cell viability. However, the overexpression of VAMP2 decreased the promotion of cell viability by miR-127-3p mimic (**P < 0.01, ***P < 0.001, Fig. 5G). Conversely, miR-127-3p inhibitor markedly reduced cell viability. Co-transfection with miR-127-3p inhibitor and si-VAMP2 significantly improved the cell viability compared with the co-transfection with miR-127-3p inhibitor and si-NC (***P < 0.001, Fig. 5H).

### miR-127-3p regulates hBMSC differentiation by inhibiting VAMP2

The role of miR-127-3p and VAMP2 on osteoblast differentiation is illustrated in Fig. 5I, J, K, L. Upregulation of miR-127-3p elevated the mRNA and protein levels of RUNX2, OCN, and OPN, whereas the promotion of differentiation by miR-127-3p mimic was reversed after co-transfection of miR-127-3p mimic and oe-VAMP2. On the other hand, downregulation of miR-127-3p reduced their levels. Co-transfection with miR-127-3p inhibitor and si-VAMP2 reversed the inhibitory effect of miR-127-3p inhibitor on differentiation (*P < 0.05, ***P < 0.001).

## Discussion

OP is a metabolic and age-related bone disease. Several miRNAs participate in OP genesis and regulation and may act as diagnostic biomarkers for OP. For example, miR-206 has been reported to regulate OP progression, and miR-181c-5p and miR-497-5p may be the biomarkers for OP diagnosis and prognosis (Ma *et al.* 2020, Lu *et al.* 2021). In our study, we observed decreased miR-127-3p expression in patients with OP. Therefore, miR-127-3p differentiate patients with OP from healthy individuals, which is consistent with previous studies. These findings suggest that miR-127-3p may be a new biomarker for OP diagnosis.

The effect of miRNAs on disease is increasingly emphasized, and research has demonstrated that about 30% of protein-coding genes in mammals are regulated by miRNAs (Matsui & Corey 2017). hBMSCs can be differentiated into osteoblasts and chondrocytes, which are broadly applied in OP and other orthopedic diseases (Zhang *et al.* 2018*a*). Various studies have reported that miRNAs may improve OP by regulating hBMSCs, among which miR-10a-3p and miR-4739 promote the osteogenic differentiation of hBMSCs and improve OP (Li *et al.* 2021, Liu *et al.* 2021). Huang *et al.* reported that miR-127-3p expression decreased in OP (Huang *et al.* 2024). Referring to previous studies, we aimed to investigate whether miR-127-3p is involved in osteogenic differentiation. We confirmed that the upregulation of miR-127-3p inhibits hBMSC apoptosis and elevates cell viability. Furthermore, the downregulation of miR-127-3p exhibits reverse outcomes. Research on differentiation in hBMSCs has revealed that transfection with miR-127-3p mimic increases RUNX2, OCN, and OPN levels. These outcomes suggest the essential role of miR-127-3p in promoting the differentiation of hBMSCs into osteoblasts.

Previous studies on orthopedic-related diseases have revealed the role of VAMP2. For example, VAMP2 has been implicated as a keystone gene in osteonecrosis of the femoral head (Zhang *et al.* 2020). In osteosarcoma, miR-185 provides rationale for treatment by reducing VAMP2 levels (Li *et al.* 2019). In addition, the involvement of VAMP2 has been found in postmenopausal OP (Pala & Denkçeken 2019). *In vitro* experiments proved that silencing VAMP2 favors hBMSC differentiation toward osteoblasts. A variety of miRNAs affect bone formation-related gene expression at the post-transcriptional level. For example, miR-215-5p was able to target XIAP to stimulate the differentiation of hBMSCs into osteoblasts (Yin *et al.* 2022). miR-127-3p targets ITGA6 to affect the proliferation and invasion of osteosarcoma cells (Wang *et al.* 2018). However, whether miR-127-3p interacts with VAMP2 to affect the hBMSC differentiation remains unknown. Our study confirmed that VAMP2 is a target of miR-127-3p and overexpression of miR-127-3p could downregulate VAMP2 levels. Simultaneous miR-127-3p and VAMP2 overexpression dramatically facilitated apoptosis and inhibited the viability and differentiation of hBMSCs, compared with the overexpression of miR-127-3p. Conversely, simultaneous downregulation of miR-127-3p and VAMP2 inhibited hBMSC apoptosis and enhanced cell viability and differentiation compared with the miR-127-3p inhibitor + si-NC group. These outcomes reveal that miR-127-3p effectively promotes hBMSC differentiation, inhibits apoptosis, and enhances bone repair through negatively regulating VAMP2. This highlights the pivotal role of miR-127-3p targeting VAMP2 in maintaining bone homeostasis, potentially providing a new direction for gene therapy for OP.

In the identification study of OP-related biomarkers, Chen *et al.* reported that the related genes were primarily enriched in lipid metabolism, calcium ion transport, and ossification pathways, including VAMP2 (Chen *et al.* 2025). In a high-throughput sequencing study to screen differentially expressed miRNAs in patients with OP, miR-127-3p was upregulated threefold in patients with OP compared with those without OP and enriched for the adipocytokine signaling pathway (Huang *et al.* 2024). The adipocytokine signaling pathway is a key upstream signaling mechanism that regulates lipid metabolism. Therefore, miR-127-3p may regulate the mechanisms underlying lipid metabolism pathways by regulating VAMP2, thereby regulating the progression of OP. Furthermore, studies have found that VAMP2, a key protein in synaptic vesicle fusion, is closely related to neurology (Wang *et al.* 2024). Nerve fibers are highly innervated by skeletal cells and play a role in information transmission, mediating sensation and regulating bone formation and regeneration (Zhao *et al.* 2025). It is hypothesized that VAMP2 expression regulated by miR-127-3p may play an important role in nerve–bone axis communication. Nevertheless, these mechanisms underlying the role of VAMP2 as a miR-127-3p target in OP need to be further explored.

The present study has several limitations. The results were only confirmed in hBMSCs. Whether miR-127-3p and VAMP2 have similar functions in animal models remains unclear. Therefore, additional experiments are warranted to confirm the role of miR-127-3p and VAMP2 in OP by *in vivo* assays. Furthermore, in the future, we will focus on the downstream regulatory network of VAMP2 to fully explore its regulatory mechanism in OP.

In conclusion, this research has highlighted the diagnostic potential of miR-127-3p, suggesting that it may serve as a new biomarker. Importantly, miR-127-3p may regulate the apoptosis, cell viability, and differentiation of hBMSCs by targeting VAMP2, which in turn slows down the OP development process and provides new ideas for treating OP in clinical settings.

## Declaration of interest

The authors declare that there is no conflict of interest that could be perceived as prejudicing the impartiality of the work reported.

## Funding

This work did not receive any specific grant from any funding agency in the public, commercial or not-for-profit sector.

## Author contribution statement

JC, JL, GW, HQ, and LY contributed to the conceptualization of the study. Data curation and formal analysis were performed by JL, GW, HQ, and LY. Funding acquisition was done by HQ and LY. Investigation was carried out by GW and HQ. Methodology was developed by JC, JL, GW, HQ and LY. Project administration was handled by HQ. Resources were provided by GW and HQ. JC, JL, GW and HQ helped with software. Supervision was provided by HQ and LY. Validation was done by GW and HQ. Visualization was performed by JC, JL and GW. GW helped in writing of the original draft. Writing of the review and editing were done by JC, JL, HQ, and LY.

## Data availability

The datasets used and/or analyzed during the current study are available from the corresponding author on reasonable request.

## Ethics approval and consent to participate

The study was performed in line with the principles of the Declaration of Helsinki. Approval was granted by the Ethics Committee of Zhucheng People’s Hospital before the study began. The written informed consent has been obtained from the participants involved.
